# An update on magnesium and bone health

**DOI:** 10.1007/s10534-021-00305-0

**Published:** 2021-05-06

**Authors:** Mariangela Rondanelli, Milena Anna Faliva, Alice Tartara, Clara Gasparri, Simone Perna, Vittoria Infantino, Antonella Riva, Giovanna Petrangolini, Gabriella Peroni

**Affiliations:** 1 IRCCS Mondino Foundation, 27100 Pavia, Italy; 2 Department of Public Health, Experimental and Forensic Medicine, Unit of Human and Clinical Nutrition, University of Pavia, 27100 Pavia, Italy; 3 Endocrinology and Nutrition Unit, Azienda Di Servizi Alla Persona “Istituto Santa Margherita”, University of Pavia, 27100 Pavia, Italy; 4 Department of Biology, College of Science, University of Bahrain, 32038 Sakhir, Bahrain; 5 Research and Development Unit, Indena, Milan Italy

**Keywords:** Magnesium, Bone, Dietary supplementation, Bone mineral density, Nutrients

## Abstract

In 2009 EFSA Panel concludes that a cause and effect relationship has been established between the dietary intake of magnesium (Mg) and maintenance of normal bone. After 2009, numerous studies have been published, but no reviews have made an update on this topic. So, the aim of this narrative review was to consider the state of the art since 2009 on relationship between Mg blood levels, Mg dietary intake and Mg dietary supplementation (alone or with other micronutrients; this last topic has been considered since 1990, because it is not included in the EFSA claims) and bone health in humans. This review included 28 eligible studies: nine studies concern Mg blood, 12 studies concern Mg intake and seven studies concern Mg supplementation, alone or in combination with other nutrients. From the various studies carried out on the serum concentration of Mg and its relationship with the bone, it has been shown that lower values are related to the presence of osteoporosis, and that about 30–40% of the subjects analyzed (mainly menopausal women) have hypomagnesaemia. Various dietetic investigations have shown that many people (about 20%) constantly consume lower quantities of Mg than recommended; moreover, in this category, a lower bone mineral density and a higher fracturing risk have been found. Considering the intervention studies published to date on supplementation with Mg, most have used this mineral in the form of citrate, carbonate or oxide, with a dosage varying between 250 and 1800 mg. In all studies there was a benefit both in terms of bone mineral density and fracture risk.

## Introduction

Magnesium (Mg) is an intracellular cation (second in abundance after potassium), ubiquitous in the human body where it is present (adult organism) in quantities of about 20–28 g: 60% is found in the bones, 39% in the intracellular compartments and about 1% in the extracellular liquids. Mg is present in almost all foods in varying concentrations. It is contained in leaf vegetables in a concentration of 30–60 mg/100 g, being in the center of the pyrrolic core of chlorophyll. Larger quantities are contained in legumes (80–170 mg/100 g), nuts (130–264 mg/100 g) and whole grains (up to 550 mg/100 g in wheat bran). More than 80% of the Mg is removed from the grain refining treatments (white bread contains only 15 mg/100 g). High quantities are present in the coffee (80 mg/100 g in the ready-to-drink) (US Department of Agriculture 2009). Dried fruit in general, potatoes and food of animal origin (meat, fish, milk and derivatives) are less rich in Mg (20–70 mg/100 g) (Carnovale and Marletta 2013). The concentration of Mg in the water is highly variable depending on its origin. The labels of the 150 bottled waters consumed in the scope of the INRAN-SCAI 2005–06 survey (Leclercq et al. 2009) show that the content varies from 1 to 109 mg/L, with an average of 15 mg/L. The bioavailability of Mg varies in the presence of specific components of the diet: phytates, calcium, phosphorus and long chain fatty acids decrease its absorption, while there are conflicting evidences about the effect of oxalic acid.

So, the potential benefits of consuming magnesium might be masked with the effects of other nutrients.

Cooking food also reduces its bioavailability (Dilworth et al. 2007), which instead increases in the presence, for example, of proteins, fructose, inulin, fruit- and galact-oligosaccharides (Roth and Werner 1979; Seelig 1981; Lönnerdal 1997; Coudray et al. 2003, 2005).

The recommended intake levels (RDA) of Mg were provided by the United States Food and Nutrition Board (Food and Nutrition Board 1997).

Several dietary surveys conducted in the United States show that many people consume less than the recommended amounts of Mg constantly. A 2013–2016 National Health and Nutrition Examination Survey (NHANES) data analysis found that 48% of Americans of all ages take less Mg from food and drink than their average needs; adult men 71 years of age and older, teenagers are more likely to show low Mg intake (US Department of Agriculture 2019).

So the dietary intake of Mg is on average insufficient, but clinical diagnosis of Mg deficiency is not simple, as symptoms associated with Mg deficiency are unspecific, and generally confounded by low consumption of other nutrients.

Unfortunately, routinely measured serum Mg levels do not always reflect total body Mg status, so normal level of serum Mg does not rule out moderate to severe Mg deficiency (Razzaque 2018).

In 2009, EFSA issued an opinion on health claims related to Mg with the diet, establishing that there is sufficient scientific evidence to indicate that dietary Mg contributes to various functions of the body, including electrolyte balance, the energy performance of the metabolism, neurotransmission and muscle contraction, including heart muscle, cell division, protein synthesis and finally the maintenance of bones and teeth (European Food Safety Authority 2009).

In particular, considering bone health, Mg has a pivotal role.

Mg deficiency might affect bone directly (by reducing bone stiffness, increasing osteoclasts and decreasing osteoblasts) and indirectly (by interfering with PTH and vit D, promoting inflammation/oxidative stress and subsequent bone loss) (Castiglioni et al. 2013).

Mg is an essential cofactor for vitamin D synthesis and activation and, in turn, can increase intestinal absorption of Mg and establish a feed-forward loop to maintain its homeostasis (Uwitonze and Razzaque 2018; Erem et al. 2019).

Given this background, the aim of this narrative review was to consider the state of the art since 2009 on relationship between Mg blood levels, magnesium dietary intake and Mg dietary supplementation (alone or with other micronutrients; this last topic has been considered since 1990, because it is not included in the EFSA claims) and bone health in humans.

## Materials and methods

The present narrative review was performed following the steps by (Egger et al. 2008) as follows:

1. Configuration of a working group: three operators skilled in clinical nutrition (one acting as a methodological operator and two participating as clinical operators).
2. Formulation of the revision question on the basis of considerations made in the abstract: “the state of the art since 2009 on the correlation between human blood Mg concentrations, daily Mg intake with food and bone mineral density and since 1990 on the correlation between Mg dietary supplementation and bone mineral density.
3. Identification of relevant studies: a research strategy was planned on PubMed (Public MedIine run by the National Center of Biotechnology Information (NCBI) of the National Library of Medicine of Bethesda (USA)) as follows: (a) Definition of the keywords (magnesium, bone health, humans, intake, supplementation, bone mineral density), allowing the definition of the interest field of the documents to be searched, grouped in quotation marks (“…”) and used separately or in combination; (b) use of: the Boolean (a data type with only two possible values: true or false) AND operator, that allows the establishments of logical relations among concepts; (c) Research modalities: advanced search; (d) Limits: time limits: papers published since 2009 for the evaluation of correlation between human blood Mg concentrations, daily dietary Mg intake and bone mineral density and since 1990 for the evaluation of the correlation between Mg dietary supplementation and bone mineral density; humans; adults; languages: English; (e) Manual search performed by the senior researchers experienced in clinical nutrition through the revision of articles on the state of the art on the correlation between human blood magnesium concentrations, daily magnesium intake with food, magnesium supplementation and bone mineral density.
4. Published in journals qualified in the Index Medicus.
5. Analysis and presentation of the outcomes: we create paragraphs about the state of the art on the correlation between human blood Mg concentrations, daily Mg intake with food, Mg supplementation and bone mineral density, and the data extrapolated from the “revised studies” were collocated in tables; in particular, for each study we specified the author and year of publication and study characteristics.
6. The analysis was carried out in the form of a narrative review of the reports. At the beginning of each section, the keywords considered and the type of studies chosen are reported. We evaluated, as is suitable for the narrative review, studies of any design which considered the state of the art on the correlation between human blood Mg concentrations, daily Mg intake with food, Mg supplementation and bone mineral density.

**Fig. 1:**
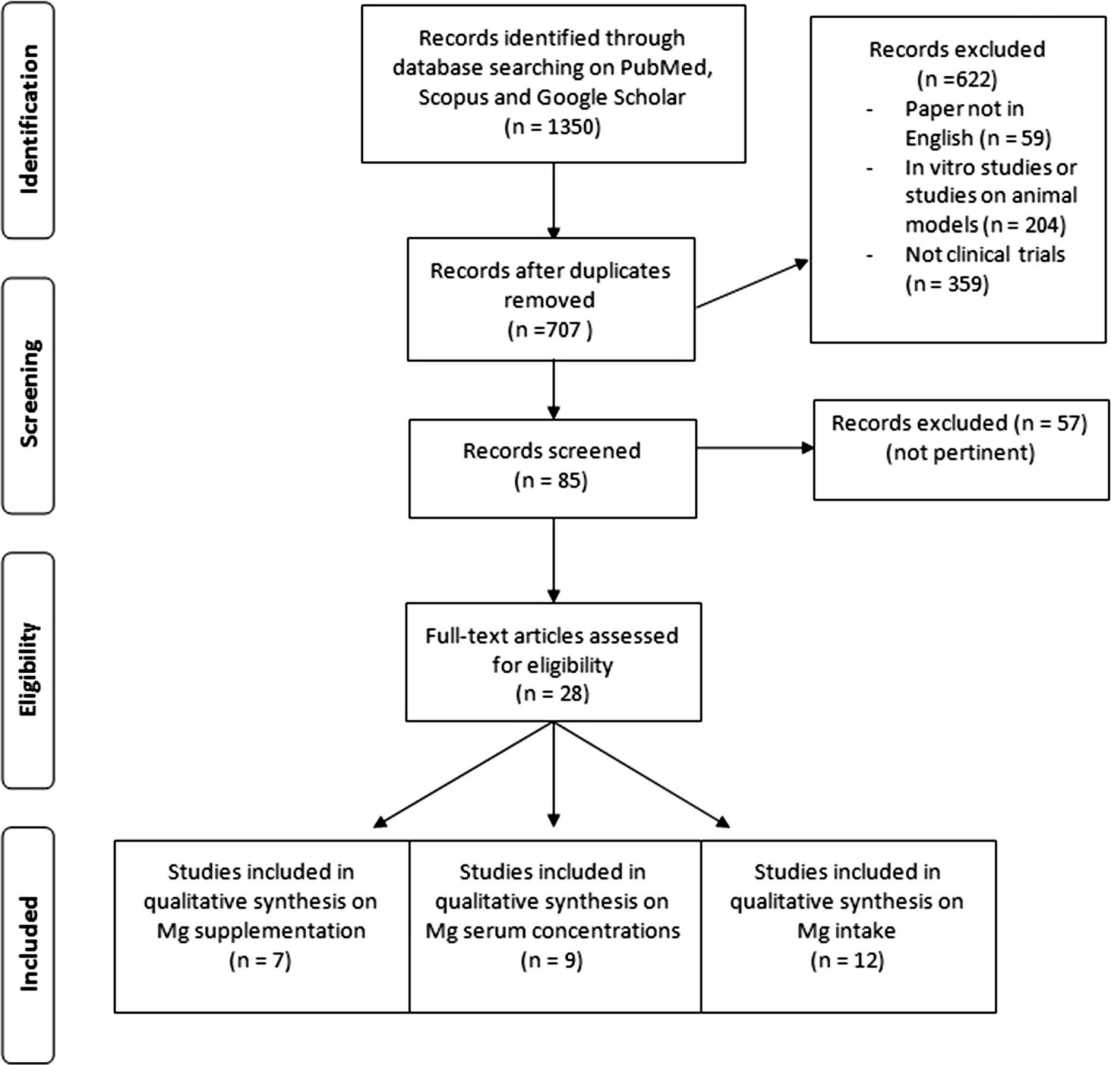
Flow chart of literature research

Figure 1 shows the flow chart of literature research.

## Results

### Serum magnesium concentration and effects on bone metabolism

This research was conducted based on the keywords: “blood magnesium” AND “bone” AND “humans”.

For the present review we have analyzed a total of nine studies: four observational studies, two case–control studies, one cross-sectional study, one longitudinal cohort study and one prospective cohort study.

The results of these seven studies have been shown in Table 1.

**Table 1:** Studies that considered blood magnesium levels

First author, year	Study design	Setting	Inclusion criteria	Exclusion criteria	Number of subjects (M-F) Mean age	Primary outcomes
Mederle et al. (2018)	Case–control study	Outpatient Department of Endocrinology of the CountyHospital,Timisoara	Women in the postmenopausal period, with lumbar or femoral neck BMD, expressed as T-score,2.5 standard deviation (SD). The control group included women in the postmenopausal period, with lumbar or femoral neck T-score. − 2 SD	Secondary causes of osteoporosis, other diseases that could influence the bone metabolism or electrolyte imbalance (especially Mg), fractures in the previous year, hormone replacement therapy, and any medication that could influence bone turnover	213 F(132 cases–81 controls)	Determine the correlations between BMD and serum levels of bone resorption markers (TRAP-5b), bone formation markers (BSAP), estradiol (E2), and Mg(2 +) ion concentrations in postmenopausal osteoporotic women as compared to healthy postmenopausal subjects
Okyay et al. (2013)	Obesrvational study	Division of Reproductive Endocrinology and Infertility, Department of Obstetrics and Gynecology at DokuzEylulUniversitySchoolof Medicine,Izmir, Turkey	Postmenopausal women between age 45 and 80 y	History of drug abuse or alcohol consumption (to drink at least ≥ 2 days per week), and highly intake of caffeinated coffee (> 2 cups per day), laboratory tests or radiography of any bone metabolism disorder	728 F	Determine the relationship between serum main minerals and postmenopausal osteoporosis
Mahdavi et al. (2015)	Observational study	Rheumatology clinic in Tabriz, Islamic Republic of Iran,	Women > 50 years old who had been no menstruation for ≥ 6 months prior to entry into the study, having no history of hormone replacement therapy, other bone disease, kidney stones, endocrine disorders or any medical conditions that could influence on the mineral status	Use of mineral supplements, having history of hormone replacement therapy, bone disease, kidney stones, endocrine disorders or any medical conditions that could influence on the mineral status	51 F(23 had osteoporosis and 28 had osteopenia)	Investigate and compare the copper, magnesium, zinc and calcium status between osteopenic and osteoporotic postmenopausal women in Tabriz, Islamic Republic of Iran
Hayhoe et al. (2015)	Case Cohort Longitudinal Study	Norfolk, UK	Men and women aged 40–82 y living in the general community	–	2328 ultrasound cohort group – 4713 fracture case-cohort group	The influence of dietary magnesium and potassium intakes, as well as circulating magnesium, on bone density status and fracture risk in an adult population in theUnited Kingdom
Sharma et al. (2016)	Observational study	TSMMedical College & Hospital, Lucknow,Uttar Pradesh, India	Postmenopausal women with 48 to 75 years	–	68 F(33 with osteoporosis and 35 with osteopenia)	The role of magnesium in osteoporosis and in osteopenia
Kunutsor et al. (2017)	Prospective cohort study	Eastern Finland	Men aged 42–61 years (a cohort of the Kuopio Ischemic Heart Disease Prospective Study) living in the city of Kuopio and its neighbouring rural communities	–	2245 M	Investigate the association of baseline serum magnesium concentrations with risk of incident fractures
Rai (2016)	Observational study	OPD Dept of Orthopedics, TSM Medical College & Hospital, Lucknow, India	Postmenopausal women 48–75 years	–	68 F(33 with osteoporosis and 35 with osteopenia)	Evaluation of magnesium role in bone homeostasis, especially in postmenopausal women with osteopenia and osteoporosis
Huang et al. (2015)	Cross-sectional study	Hospital clinic of Central Taiwan	CKD patients not receiving dialysis	–	56 (27 with Diabetes and 29 without Diabetes)	Investigate the impact of serum Mg on bone mineral metabolism in chronic kidney disease (CKD) patients with or without diabetes
Elshal et al. (2012)	Case–control study	Outpatient clinic of the university hospital of	Adults with an age range 20–40 years with sickle-cell anaemia in steady-state and race-matched healthy blood donors	Use of steroids, had anorexia nervosa, hyperthyroidism, chronic obstructive pulmonary disease, liver disease, inflammatory bowel disease, or had deranged renal functions (serum creatinine > 2.5 mg/dl)	60 with sickle-cell anaemia (34 F – 26 M) and 40 healthy blood donors as controls (22 F – 18 M)	Investigate whether serum Mg levels may have an impact on bone mineral density in sickle-cell anaemia

First author, year	Micronutrient serum concentration osteoporosis	Micronutrient serum concentration osteopenia	Micronutrient serum concentration normal	Micronutrient serum reference value	% subjects < reference value	Results
Mederle et al. (2018)	1.76 ± 0.06 mg/dl	2.14 ± 0.14 mg/dl	–	1.6–2.4 mg/dL	–	Osteoporotic patients showed significantly lower concentrations of serum Mg(2 +) than the control group. Mg(2 +) levels correlated positively with BMD values (r = 0.747, P,0.0001)
Okyay et al. (2013)	45–59 y: 0.86 ± 0.1 mg/dl (osteo L1-L4), 0.84 ± 0.16 mg/dl (osteo total femur), 0.86 ± 0.18 mg/dl (osteo femoral neck)—60–80 years: 0.85 ± 0.1 mg/dl (osteo L1-L4), 0.85 ± 0.14 mg/dl (osteo total femur), 0.86 ± 0.16 mg/dl (osteo femoral neck)	–	45–59 y: 0.89 ± 0.1 mg/dl (non osteo L1-L4), 0.89 ± 0.17 mg/dl (non osteo total femur), 0.89 ± 0.16 mg/dl (non osteo femoral neck)—60–80 years: 0.94 ± 0.1 mg/dl (non osteo L1-L4), 0.91 ± 0.18 mg/dl (non osteo total femur), 0.91 ± 0.17 mg/dl (non osteo femoral neck)	–	45–59 y: 47,1%—52,9% (osteo – non osteo L1-L4)/29,4%—70,6% (osteo – non osteo total femur)/32,4%—67,6% (osteo – non osteo femur neck)	Low serum magnesium levels had significant association with osteoporosis of L1–L4 spines and total femur
Mahdavi et al. (2015)	0.76 ± 0.02 mmol/l	0.77 ± 0.01 mmol/l	–	–	40,4%	40.4% of patients had serum magnesium level lower than normal range
Hayhoe et al. (2015)	–	–	–	0.7–1.0 mmol/l	–	Statistically significant trends in fracture risk in men across serum magnesium concentration groups were apparent for spine fractures (P = 0.02) and total hip, spine, and wrist fractures (P = 0.02)
Sharma et al. (2016)	1.95 ± 0.44 mg/dl	2.22 ± 0.42 mg/dl	–	1.9–2.5 mg/dl	–	The serum concentration of magnesium was lower in osteoporosis group and the result was statistically significant (< 0.05)
Kunutsor et al. (2017)	–	–	–	1.8–2.3 mg/dl	6% of subject with fractures	Low serum magnesium is strongly and independently associated with an increased risk of fractures
Rai (2016)	1.95 ± 0.44 mg/dl	2.22 ± 0.42 mg/dl		–	–	The serum concentration of magnesium (1.95 ± 0.44 vs. 2.22 ± 0.42) was lower in osteoporosis group, and the result was statistically significant (< 0.05)
Huang et al. (2015)	–	–	–	1.82–2.31 mg/dl	10,7%	The lower serum Mg subgroup showed a higher incidence of osteoporosis than the moderate and higher serum Mg subgroups did (66.7%, 39.4%, and 29.4%, resp.)
Elshal et al. (2012)	–	–	–	0.7–1.2 mmol/l	33,3% of subjects with sickle-cell anaemia	The serum magnesium was found to be associated positively with serum calcium (Ca), PTH and Osteocalcin (*r* = 0.585; *r* = 0.436; *r* = 0.351 respectively, all at *p* < 0.05), and negatively with PO4 (*r* = –0.312; *p* < 0.05). Hypo-Magnesium patients had significantly lower BMD and T-score at all evaluated sites (L2, L4, and WB) than norm-Magnesium patients and controls (all *p* < 0.05)

#### Magnesemia and bone mineral density

With regard to the serum concentration of Mg, most of the studies has focused on postmenopausal women. In this regard, Mederle et al. conducted a study in 2018 to investigate the correlation between bone mineral density (BMD) and serum Mg levels in 132 post-menopausal osteoporotic women compared with 81 healthy (non-osteoporotic) women, always in post-menopause. Osteoporotic women showed significantly lower concentrations of serum Mg compared to the control group (1.76 ± 0.06 mg/dl compared to 2.14 ± 0.14 mg/dl), while remaining in the reference range (1.6–2.4 mg/dl), moreover i Mg levels were positively correlated with BMD values (Mederle et al. 2018). Another study on postmenopausal women is that carried out by Okyay et al. in 2013, in which the relationship between the serum concentrations of different minerals was assessed, including Mg and the presence of osteoporosis. The 728 women enrolled were then divided into 2 groups, according to the presence or absence of osteoporosis, and the results of the analysis carried out found a significant association between low serum Magnesium (Mg) values and the presence of both lumbar (L1—L4) and osteoporosis femoral. In this study it was also highlighted that, between 45 and 59 years, 47.1% of osteoporotic women at the level of L1-L4, 29.4% of osteoporotic women at the level of the whole femur and 32., 4% of osteoporotic women at the neck of the femur have a serum Mg value lower than the reference range (Okyay et al. 2013). Moreover, the group of Mahdavi et al. wanted to examine the serum concentration of Mg, Zinc, Copper and Calcium in 51 women: 23 osteoporotic and 28 osteopenic. Specifically, for Mg it was shown that 40.4% of women had a lower serum concentration than the reference ranges, without statistically significant differences between the 2 groups (Mahdavi-Roshan et al. 2015). Other analysis on the relationship between serum Mg concentration and the presence of osteopenia and osteoporosis have been carried out: a study carried out in India has observed that serum Mg in women with osteoporosis (1.95 ± 0.44 mg/dl) was significantly lower than to women with osteopenia (2.22 ± 0.42 mg/dl), however remaining within the reference ranges (1.9–2.5 mg/dl) (Sharma et al. 2016). Also in India, a further study on 68 women, including 33 osteoporotic and 35 with osteopenia, observed that the serum Mg concentration was lower in the osteoporosis group (1.95 ± 0.44 mg/dl) compared with osteopenic women (2.22 ± 0.42 mg/dl) in a statistically significant way (Rai and Sharma 2016).

#### Magnesiemia and fracturing risk

Moreover, it is important to evaluate the role of Mg in fracture risk, both in women and men. In this regard, a UK cohort study, analyzing subjects of both sexes belonging to the EPIC-Norfolk study, investigated the influence of serum Mg on bone density, assessed by means of a bone analyzer ultrasound, and fracture risk. The analysis showed statistically significant trends in the risk of fracture in men, especially for spinal (P = 0.02), hip and wrist fractures (P = 0.02) (Hayhoe et al. 2015).

Another cohort study carried out in Finland, analyzing subjects from the KIHD study, wanted to study the association of serum magnesium concentrations with the risk of fractures in adult subjects of both sexes. Considering a reference range of 1.8–2.3 mg/dl, 6% of subjects with fractures were found to have a lower serum Mg value; moreover, the Mg concentration was strongly associated with a high risk of fractures (Kunutsor et al. 2017).

In addition to "healthy" subjects, it can be useful to evaluate the Mg values in the blood also in subjects with pathologies that in some way can interfere with a normal bone turnover. Chronic kidney disease causes progressive renal function decline over time. Serum Mg levels may increase with renal functional decline (Navarro-González et al. 2009) and this may possibly be harmful to bone health (Castiglioni et al. 2013). Huang and colleagues, analyzing 56 patients with chronic kidney disease, not yet on dialysis, found that 10.6% of the subjects had a serum Mg value lower than the reference range (1.82–2.31 mg/dl) (Huang et al. 2015). The different pathological conditions of the bone, including osteopenia and osteoporosis, are also a frequent cause of morbidity in sickle cell anemia. Mg regulates some important biological processes in bone remodeling and participates in the pathophysiology of erythrocyte sickle. 60 adult subjects—with an age range from 20 to 40 years—with sickle cell anemia were analyzed by evaluating their serum Mg concentration: 33.3% were found to be deficient. Furthermore, there was a positive association between serum Mg, calcium, parathyroid hormone and osteocalcin, and subjects with hypomagnesaemia were found to have a lower bone mineral density, both lumbar and whole body, compared to subjects with normal serum values (Elshal et al. 2012).

In conclusion, all studies published since 2009 agree in confirming that subjects with hypomagnesaemia were found to have a lower bone mineral density.

### Dietary intake of magnesium and effects on bone metabolism

This research was conducted based on the keywords: “dietary magnesium intake” AND “bone” AND “humans”. It have been considered studies since 2009.

For the present review we have analyzed a total of 12 studies: five prospective studies, two cross-sectional studies, one longitudinal cohort study, two prospective cohort studies, one observational study and one cross-sectional cohort study.

The results of these seven studies have been shown in Table 2.

**Table 2:** Studies that considered magnesium dietary intake

First author, year	Study design	Setting	Inclusion criteria	Exclusion criteria	Number of subjects (M-F) mean age	Lowest quintile intake/RDA or EAR
Wright et al. (2019)	Prospective Cohort study	North-West University, Potchefstroom campus	Postmenopausal urban black South African women from PURE-SA-NWP, that underwent measurements of distal radius BMD, dual-energy X-ray absorptiometry (DXA), and had their blood profiles done in both 2010 and 2012	–	144 F	−/265 mg/d (EAR)
Onchard et al. (2014)	Prospective Cohort study	40 clinical centers throughout theUnited States	Postmenopausal women enrolled in the Women’s Health Initiative Observational Study	Missing data on magnesium or other model covariates	73,684 F	< 206.5 mg/d
Abrams et al. (2014)	Prospective study	GeneralClinical Research Center (GCRC) of Texas Children’s Hospital inHouston, TX, USA	Healthy subjects with 4.0 to 8.9 years of age at the time of starting the study not using any medications or multivitamins/multiminerals	Body mass index (BMI) Z‐score > 2.0	50 (28 F + 22 M)	−/110 mg/d (EAR)
Matias et al. (2012)	Prospective study	Faculty of Human Kinetics, Technical University ofLisbon	Elite swimmers, males and females, with minimum period of activity of approximately six years; > 10 h training per week; negative test outcomes for performance enhancing drugs; not taking any medications or dietary supplements	–	17 (9 F + 8 M)	−/400 mg/d (RDA)
Esterle et al. (2009)	Prospective study	Junior high schools and at theUniversity of Caen	Healthy adolescent girls and young women (12 to 22 years old)	–	192 F	−/360 mg/d (RDA)
Gunn et al. (2014)	Cross sectional study	Community dwelling from the Auckland, Hawke’s Bay and Manawatu regions in New Zealand	Healthy, postmenopausal women aged 50–70 years	Any known significant health condition or regular use of medication which could affect bone or inflammation including HRT, NSAID’s and proton pump inhibitors	142 F	−/320 mg/d (RDA)
Kim et al. (2011)	Prospective study	Department of Food and Nutrition, Kangwon National University, Gangwon-do	Healthy females aged 19–25 years	Taken any medications or nutritional supplements	484 F	−/280 mg/d (EAR)
Farrell et al. (2009)	Cross-sectional study	University of Arizona, Department of Nutritional Sciences	Cohorts (Fall 1995–Fall 1997) of postmenopausal women from the first year of the Bone Estrogen Strength Training (BEST), a blocked- randomized, clinical trial, with: 12-month measurements of BMD at the 5 sites of interest (lumbar spine L2–L4 (1.130.16 g/cm3), femur trochanter (0.75 ± 0.11 g/cm3), femur neck (0.88 ± 1.12 g/cm3), Ward’s triangle (0.76 ± 1.14 g/cm3), and total body (1.11 ± 0.08 g/cm3)	–	244 F	−/320 mg/d (RDA)
Veronese et al. (2017)	Prospective study	Four clinical sites in the US (Baltimore, MD; Pittsburgh, PA; Pawtucket, RI; and Columbus, OH)	Subjects enrolled in the Osteoarthritis Initiative (OAI) database, at high risk of knee OA	–	3765 (1577 M – 2071 F)	< 205 mg/d M—< 190 mg/d F
Welch et al. (2017)	Cross-sectional Cohort study	United Kingdom (UK) Biobank cohort	People aged 37–73 years	Subject without dietary or other relevant missing data, non-white ethnicity, pregnant women, those with a grip strength of zero, those with extremes of FFM, BMD, Mg, energy, protein, EI:EER, or BMI	156,575 (36,118 M – 40,441 F in bone analysis)	238 ± 37 mg/d M—217 ± 34 mg/d F
Hayhoe et al. (2015)	Case Cohort Longitudinal Study	Norfolk District (UK)	Men and women aged 40–82 y living in the general community	–	2328 ultrasound cohort group – 4713 fracture case-cohort group	218 ± 31 mg/d M—189 ± 26 mg/d F for ultrasound cohort group and 209 ± 31 mg/d M—175 ± 25 mg/d F for fracture case-cohort group
Mahdavi et al. (2015)	Observational study	Rheumatology clinic in Tabriz, Islamic Republic of Iran,	Women > 50 years old who had been no menstruation for ≥ 6 months prior to entry into the study, having no history of hormone replacement therapy, other bone disease, kidney stones, endocrine disorders or any medical conditions that could influence on the mineral status	Use of mineral supplements, having history of hormone replacement therapy, bone disease, kidney stones, endocrine disorders or any medical conditions that could influence on the mineral status	51 F(23 had osteoporosis and 28 had osteopenia)	325 mg/d

First author, year	% subject in lowest quintile intake/% subject < RDA or EAR	Highest Quintile intake	% subject in highest quintile intake	Primary outcomes	Results
Wright et al. (2019)	−/21,1%	–	–	Investigate the association between nutrient intake and dietary patterns (exposures) with changes in bone turnover and BMD (bone health outcomes) in postmenopausal urban black South African women	Dietary magnesium negatively correlated with CTx-1 in 2012 (r = -0.21, p = 0.02). The baseline CTx-1 and dietary magnesium intake predicted 22% of the variance in percentage change of CTx-1 over two years (p < 0.001). The magnesium intake predicted short-term bone resorption over two years
Onchard et al. (2014)	19.7%	≥ 422.5 mg/d	20.2%	Magnesium intake as a risk factor for osteoporotic fractures and altered bone mineral density (BMD)	Baseline hip BMD was 3% higher (P < 0.001), and whole body BMD was 2% higher (P < 0.001), in women who consumed > 422.5 compared with < 206.5 mg mg/d of magnesium
Abrams et al. (2014)	–	–	–	Mg intake or absorption and is related with bone mineral content (BMC) or bone mineral density (BMD) in children	Mg intake and total Mg absorption were significantly associated with both total body BMC and BMD
Matias et al. (2012)	–	–	–	Mg intake mediates the association between BMD and LST in elite male and female swimmers	Mg intake was a significant, independent predictor of BMD, with a significant increase of 24% in the R2 of the initial predictive model
Esterle et al. (2009)	Magnesium intakes were lower than the recommended dietary allowances in all the participants	–	–	Identify dietary foods and nutriments associated with lumbar bone mineral content (BMC) and bone mineral density (BMD) in adolescent girls	After menarche, BMC, BMD, serum IGF-1, and serum PTH were tightly associated with phosphorus, magnesium, protein, and energy from milk (p < 0.01)
Gunn et al. (2014)	–	–	–	Investigate diet and nutrition-related factors associated with bone loss in a group of postmenopausal (PM) women	Positive correlation with Mg intake and BMD and Procollagen type I N propeptide (p < 0.05)
Kim et al. (2011)	79,96%	–	–	Assess Mg intake in early adult stage women and examine its relationship on bone quality	The level of Mg intake per 1,000 kcal showed significant correlation to the speed of sound in the calcaneus (r = 0.110, p < 0.05) after adjustment for age, BMI, and percent body fat
Farrell et al. (2009)	–	–	–	Diet records (DR) and Food Frequency Questionnaires (FFQ), assessing the same year of dietary intake, provide equivalent estimates of nutrient intakes when determining the associations of dietary nutrient intakes with BMD in healthy, post-menopausal women	Iron and magnesium were significantly associated with all BMD sites regardless of the dietary assessment method used
Veronese et al. (2017)	20% M – 19,9% F	> 398 mg/d M—> 373 mg/d F	20% M – 19,8% F	The effect of higher Mg intakes on the onset of fractures in a large cohort of American men and women involved in the Osteoarthritis Initiative	Men (HR = 0.47; 95%CI: 0.21–1.00, p = 0.05) and women (HR = 0.38; 95%CI: 0.17–0.82, p = 0.01) in the highest quintile reported a significant lower risk of fracture. Women meeting the recommended Mg intake were at an 27% decreased risk of future fractures
Welch et al. (2017)	20,6% M—19,9% F	532 ± 87 mg/d M –476 ± 75 mg/d F	20,5% M—19,9% F	Associations between dietary Mg intake and musculoskeletal health (skeletal muscle mass, hand grip strength and heel bone density) in middle and younger older aged men and women	Significant inter-quintile differences across intakes of magnesium existed in men and women, respectively, 2.9% and 0.9% for BMD
Hayhoe et al. (2015)	–	466 ± 73 mg/d M—383 ± 58 mg/d F for ultrasound cohort group and 460 ± 75 mg/d M—373 ± 59 mg/d F for fracture case-cohort group	–	The influence of dietary magnesium and potassium intakes, as well as circulating magnesium, on bone density status and fracture risk in an adult population	Statistically significant positive trends in calcaneal BUA for women but not men were apparent across increasing quintiles of magnesium plus potassium (Mg + K) z score intake (P = 0.03). Reduced hip fracture risk in both men and women was evident for individuals in specific Mg + K z score intake quintiles compared with the lowest
Mahdavi et al. (2015)	–	–	–	Investigate and compare the copper, magnesium, zinc and calcium status and intake between osteopenic and osteoporotic postmenopausal women in Tabriz, Islamic Republic of Iran	The mean dietary intake of magnesium, zinc and calcium in post-menopausal women with low bone density were significantly lower than recommended dietary allowance

For human studies, the investigations on food intake were carried out mostly thanks to the use of questionnaires (Food Frequency Questionnaire mainly) and the subjects analyzed were mainly pre-or post-menopausal women. A recent evaluation of a cohort of 144 postmenopausal black women from the PURE-SA-NWP study (Teo et al. 2009) analyzed the association between intake and dietary patterns with changes in bone turnover and bone mineral density. Mg intake was lower than the estimated average requirement (265 mg/day) for 21.1% of women and statistically correlated negatively with type I collagen C-Telopeptide, a specific marker of bone resorption (Wright et al. 2019). Furthermore, the bone mineral density of the hip and the whole body were significantly higher, respectively by 3% and 2%, in women who with intake > 422.5 mg/day compared to intake of Mg < 206.5 mg/day (Orchard et al. 2014). The positive association between bone mineral density and Mg intake was also found in another analysis conducted on 142 post-menopausal women: in addition to this data, it was also possible to observe the positive association, statistically significant, between Mg and Propeptide type I Procollagen, a marker of collagen formation during bone formation (Gunn et al. 2014). In addition to the questionnaires already mentioned, there are also various methods of evaluating the food intake; the most widely used is certainly that of the food diary, which consists in recording the food taken for 3 consecutive days, one of which at the weekend. 244 post-menopausal women used both this method and the Food Frequency Questionnaire, to compare the intake in relation to their bone mineral density: in this case, in addition to Mg, also Iron was found to be positively associated with bone density regardless of the dietary assessment method used (Farrell et al. 2009). Regarding the recommended daily dose of Mg, Mahdavi and collaborators have found that, in post-menopausal osteoporotic and osteopenic women, the average dietary intake of Mg, Zinc and Calcium was significantly lower, confirming again once the relationship between these minerals and good bone maintenance (Mahdavi-Roshan et al. 2015).

As with the serum concentration, also in men it is important to evaluate the Mg intake and its relationship with bone density. A study analysis aimed to evaluate 3765 subjects of both sexes, enrolled in the Osteoarthritis Initiative (OAI) database of 4 American states, considering the association between their Mg intake and fracturing risk: men and women resulting in the quintile of intake higher (> 398 mg/day men and > 373 mg/day women) reported a significantly lower risk of fracture; in addition, women with an intake equivalent to the recommended dose of Mg found a 27% reduced risk of future fractures. Despite this, the intake of 19.9% of women and 20% of men was lower than the lowest quintile (< 205 mg/day men and < 190 mg/day women), and therefore with a greater risk of developing fractures or to reduce bone density (Veronese et al. 2017). With reference to the intake quintiles, similar results were also found in the UK population, in an analysis that involved subjects of both sexes with a fairly wide age range (37–73 years): specifically the intake of Mg and 19.9% of women and 20.6% of men were lower than the lowest quintile (238 ± 37 mg/day men and 217 ± 34 mg/day women). In addition to this, significant differences were also found between the quintiles of Mg for bone mineral density, in men and women, of 2.9% and 0.9% respectively, thus further confirming the relationship between Mg and bone (Welch et al. 2017). Regarding fractures, a UK cohort study, analyzing subjects of both sexes belonging to the EPIC-Norfolk study, investigated the influence of Mg and Potassium intake on bone density, evaluated using an ultrasound bone analyzer and fracture risk. In this regard, positive, statistically significant trends in attenuation of calcaneal broadband ultrasound for women, but not for men, have been highlighted, through the increase in Mg + potassium quintiles. The reduced risk of hip fracture, both in men and in women, was instead shown in higher quintiles of Mg + Potassium than the lowest (Hayhoe et al. 2015).

A correct assessment of the Mg intake in relation to bone is important to implement also in young subjects, such as children or adolescents. A study conducted on 192 teenage girls from the University of Caen, France, showed that their Mg intake was lower than the recommended daily dose (360 mg/day), also observing that, after menarche, bone mineral density and the parathyroid hormone is closely associated with the intake of Mg, Phosphorus and milk proteins (Esterle et al. 2009). The relationship between Mg intake and correct bone development was also investigated in North Korean adolescents: out of 484 girls analyzed, the intake of 79.96% of the sample was lower than the recommended average requirement (280 mg/day). Furthermore, the intake level of Mg per 1,000 kcal showed a significant correlation with the speed of sound in the heel, detected by the instrument used for bone mineral density (Kim et al. 2011).

Male adolescents were also subjected to several analyses. Abrams and collaborators investigated the intake of Mg and its relationship with bone mineral density and bone mineral content on adolescents/children of both sexes: the intake of Mg and its absorption were significantly associated with both density and mineral content (Abrams et al. 2014). The mother's diet during pregnancy is also an important factor to consider in order to better evaluate the baby's bone mineral density.

Physical activity is also fundamental for bone mineralization, in addition, the loss of bone mass was accelerated in subjects with low Mg intake. In this regard, a study carried out in Portugal has evaluated whether, in 17 elite swimmers of both sexes, the Mg intake could have an association with bone mineral density; to reinforce this thesis, the intake of Mg was actually a significant predictor of both bone density and lean tissue, thus confirming that young athletes engaged in low-impact sports should pay particular attention to the intake of Mg, given its potential role in the acquisition of bone mineral mass during growth (Matias et al. 2012).

In conclusion, various dietetic investigations performed since 2009 have shown that many people (about 20%) constantly consume lower quantities of Mg than recommended; moreover, in this category, a lower bone mineral density and a higher fracturing risk have been found. These results have been demonstrated in both the elderly and young people.

### Magnesium dietary supplements alone or in combination with other nutrients

This research was conducted based on the keywords: “ magnesium supplementation” AND “bone” AND “humans”. It have been considered studies since 1990.

For the present review we have analyzed a total of 7 studies: 3 case–control studies, 1 retrospective study, 1 randomized controlled trial, 1 prospective, placebo-controlled randomized double blind trial and 1 double blind, placebo-controlled trial.

The results of these seven studies have been shown in Table 3.

**Table 3:** Studies that considered Magnesium supplementation

First author, year	Study design	Setting	Inclusion criteria	Exclusion criteria	Intervention	Parallel treatments
Elsinger et al. (1993)	Retrospective study		Osteoporotic women			
Stending-Lindberg et al. (1993)	Case- control	Back Rehabilitation Unit of Ichilov Hospital	Postmenopausal women with musculoskeletal pain of non-malignant origin and an initial bone density value below the reference range (≤ 1.19 g/cm3)	Diseases which preclude magnesium treatment (kidney disease, hypotension, A-V block or myasthenia gravis)	At beginning two Mg(OH)_2_ tablets (250 mg magnesium); The dosage was increased according to individual tolerance levels, to reach a maximum of two tablets three times daily (750 mg magnesium). The maximum dose was given for 6 months, followed by a maintenance dose of two tablets once daily (250 mg magnesium) for another 18 months	Assessment of bone density on two consecutive years during the period of the study, but no treatment
Carpenter et al. (2006)	Prospective, placebo-controlled, randomized, one-year double-blind trial	Clinical Research Centers at Yale University School of Medicine	Caucasian ethnicity, a ratio of weight-to-height between the third and 97th centiles, and the absence of bone disease	Scoliosis, onset of menses, use of chronic medications (retinoids, thyroid hormone, GH, glucocorticoids, oral contraceptives, anticonvulsants, diuretics, or supplements providing pharmacological dosages of vitamins A or D)	Twice daily in a capsule containing powdered magnesium oxide (300 mg of elemental Mg per day)	Encapsulatedmethylcellulose powder
Aydin et al. (2010)	Randomized controlled trial	Marmara University Medical School	Postmenopausal osteoporotic women with normal renal and hepatic function without use of drug that affect bone metabolism	Chronic systemic or bone disease or had a history of smoking, drug, or alcohol abuse	Daily oral dose of 1,830 mg magnesium citrate in the form of magnesium pastilles	Any treatment
Dimai et al. (1998)	Case- control study	University Hospital of Graz Medical School	Men, from 27–36 yr of age	Smokers, history of drug or alcohol abuse. Abnormal values of serum parameters of hepatic and renal functions or abnormal serum electrolytes and iPTH levels	Daily oral dose of 15 mmol Mg in the form of powder, containing 670 mg magnesium carbonate precipitate (equivalent to 169 mg Mg) and 342 mg magnesium oxide (equivalent to 196 mg), dissolved in 250 mL drinking water, taken in the early afternoon with a 2-h fasting period before and after the Mg intake	Glass of water daily in the afternoon with 2-h fasting
Abraham et al. (1990)	Case–control study	Menopause clinic	Postmenopausal women with hormonal replacement therapy	–	A complete supplement containing 500 mg of calcium as the citrate salt and 200 mg of magnesium as the oxide; six tablets/day	Dietary advice but chose not to take the supplement
Wood et al. (2001)	Double-blind, placebo-controlled trial	Center for Pediatric Nutrition Research, Department of Pediatrics, University of Utah, Salt Lake City, UT	Preadolescent girls (age 12, Tanner Stage 2)	–	Chewable vitamin/mineralsupplement: four tablets per day provided 800 mg/d elemental calcium (as calcium citrate and carbonate), 400 mg/d elemental magnesium (as magnesium citrate and oxide), and 400 IU/d vitamin D3	Placebo supplement containing no vitamins or minerals

First author, year	Number of subjects (M-F) If only	Duration of the intervention	Primary outcomes	Secondary outcomes	Results
Elsinger et al. (1993)	53 F	14–22 months	Effects of silicon, fluoride, etidronate and magnesium on bone mineral density		Comparisons between BMD of controls (n = 16) and treated groups over a 14–22 month period showed that fluoride (n = 10) induced a significant (P < 0.05) increase in vertebral and a slight decrease in femoral BMD, whereas silicon (n = 8) induced a significant (P < 0.05) increase in femoral BMD. Etidronate (n = 13) and, to a lesser extent, magnesium (n = 6), induced a slight although statistically non-significant increase in vertebral BMD
Stending-Lindberg et al. (1993)	31 F(case)—23 F (controls, without any symptoms that refused the treatment)	2 years	Effect of magnesium treatment on trabecular bone density in postmenopausal osteoporotic women	–	The mean bone density of the responders increased significantly both after one year (P < 0.00 1) and after 2 years (P < 0.02), while in untreated controls. the mean bone density decreased significantly (P < 0.001). Magnesium therapy prevented fractures and resulted in significant Increase of bone density In 71 per cent and arrest of bone loss in another 16 per cent of the patients
Carpenter et al. (2006)	23 F (Mg treated) – 27 F (placebo)	12 months	Identification of effect size and determination of compliance with Mg supplements in adolescents	Mg supplementation’s safety and acceptability for adolescents	Significantly increased accrual (P = 0.05) in integrated hip BMC occurred in the Mg supplemented vs. placebo group. Lumbar spinal BMC accrual was slightly (but not significantly) greater in the Mg-treated group. Compliance was excellent; 73% of capsules were ingested as inferred by pill counts
Aydin et al. (2010)	20 F (10 magnesium-supplemented and 10 unsupplemented)	30 days	Evaluate the short-term effects of daily oral magnesium supplementation on biochemical markers of bone turnover in postmenopausal osteoporotic women	–	Thirty consecutive days of oral magnesium supplementation caused significantly decrease in serum iPTH levels in the Mg-supplemented group (p < 0.05). Serum osteocalcin levels were significantly increased (p < 0.001) and urinary deoxypyridinoline levels were decreased (p < 0.001)
Dimai et al. (1998)	24 M (12 supplement + 12 controls)	30 days	Oral supplementation of a moderate dose of Mg suppresses bone turnover rates	–	The Mg supplementation significantly reduced the serum iPTH level. Mg supplementation also reduced levels of both serum bone formation and resorption biochemical markers after 1–5 days
Abraham et al. (1990)	26 F (19 case – 7 controls)	6–12 months	Validity of magnesium instead of calcium for the management of primary postmenopausal osteoporosis (PPMO)	–	A nonsignificant increase, 0.7%, in the mean Bone Mineral Density of the seven patients receiving hormonal therapy and dietary advice was observed as compared to a mean increase of 11% in the 19 women receiving the supplements
Wood et al. (2001)	81 F (38 supplement – 43 placebo)	1 year	Assessing the impact of a daily calcium, magnesium, and vitamin D supplement on bone development and bone mineralization in preadolescent girls	–	Girls receiving the calcium, magnesium, and vitamin D supplement showed a net gain in trabecular bone mineral density of 1.41% over baseline, while girls in the placebo group showed a net decline of -0.94% (p = 0.005). Percent gains in trabecular bone mineral content after 12 months of supplementation were also greater in the active treatment group than in placebo (5.83% versus 0.69% respectively)

The effect of Mg supplementation on bone mass has not been extensively studied. New research is therefore being developed in order to evaluate the effects of different nutritional supplements on bone health, both in subjects with osteoporosis and in "healthy" subjects.

The studies relating to the supplementation of Mg for the bone mainly concern postmenopausal women. A randomized controlled clinical trial sought to analyze the short-term effects of daily oral supplementation of Mg on biochemical markers of bone turnover in 20 post-menopausal osteoporotic women. Divided into 2 groups, 10 women received a treatment for 30 days, which consisted of a daily oral dose of 1,830 mg of magnesium citrate in the form of magnesium tablets, while the other 10, not receiving any supplementation, were considered as controls. In the Mg supplemented group, a significant reduction in serum parathyroid hormone levels was observed; a significant increase in serum osteocalcin levels and a significant decrease in urinary deoxypyridinoline levels (Aydin et al. 2010). Mg supplementation in osteoporotic postmenopausal women was also evaluated in relation to trabecular bone density: 54 women therefore participated in an Israeli case–control study, lasting 2 years. 31 women at the beginning of the study received two Mg tablets (250 mg of Mg); the dosage was then increased based on individual tolerance levels, to reach a maximum of two tablets three times a day (750 mg of Mg). The maximum dose was administered for 6 months, followed by a maintenance dose of two tablets once a day (250 mg of magnesium) for an additional 18 months. 23 postmenopausal women with symptom-free osteoporosis who refused treatment were instead analyzed as controls. The average bone density of treated women increased significantly after one year and after 2 years, while the average bone density decreased significantly in untreated women. Mg therapy prevented fractures and resulted in a significant increase in bone density in 71% of women and stopping bone loss in 16% (Stendig-Lindberg et al. 1993). This increase in bone density was also found in another study, in which 53 osteoporotic women, divided according to the intake of Silicon (8 women), Fluoride (10 women), Etidronate (13 women) and Mg (6) supplements women), were compared with 16 women without treatment, for a period of 14–22 months. It was therefore possible to observe how Mg induced a slight but statistically insignificant increase in vertebral bone mineral density (Eisinger and Clairet 1993).

Mg supplementation in menopausal women has also proved more effective in a combination with other minerals. In this regard, a study analyzed the effect on mineral density of the calcaneus bone in post-menopausal women, with hormone replacement therapy in place, of a supplement of Mg and Calcium for 6–12 months. 26 women were then divided into 2 groups: one, consisting of 19 women, with the intake of a complete supplement containing 500 mg of calcium as citrate salt and 200 mg of Mg as oxide, with a dosage of 6 cp/day, the other, with 7 women, followed with dietary advice but who have not chosen to take the supplement. An insignificant increase—of 0.7%—in the average bone mineral density of the 7 women who underwent dietary advice was observed while in the 19 women who took the supplements an average increase of 11% was observed (Abraham and Grewal 1990).

Therefore confirmed the importance of Mg supplementation in menopausal women, it is useful to investigate how Mg could be useful in young subjects, even if not deficient in Mg in the blood. In Austria, 24 men with an age range of 27–36 years participated in a 30-day case–control study with the aim of evaluating how oral supplementation of a moderate dose of Mg can suppress bone turnover rates. The subjects were divided into 2 groups: 12 men took a daily oral dose of 15 mmol of Mg in the form of a powder, containing 670 mg of Mg carbonate precipitate (equivalent to 169 mg of Mg) and 342 mg of oxide of Mg (equivalent to 196 mg), dissolved in 250 ml of drinking water, taken in the early afternoon, with a fasting period of 2 h before and after taking, while the other 12, considered as controls, took a glass of water every day in the afternoon after a 2-h fast. In this case, supplementation with Mg significantly reduced the serum level of the parathyroid hormone. Mg supplementation also reduced the levels of biochemical markers of both serum formation and reabsorption after 1–5 days (Dimai et al. 1998).

Considering the role of Mg also in the correct bone growth, analysis were conducted on teenagers, especially female. Carpenter and collaborators conducted a prospective, double-blind, randomized, placebo-controlled, one-year study in which 50 teenage girls were divided into 2 groups: 23 of them had to take 1 cp twice a day containing Mg oxide powder (300 mg of elemental Mg per day), while other 27 of methylcellulose powder encapsulated with the same dosage. In the group with Mg supplementation, there was a significant increase in hip bone mineral content. Lumbar spinal bone mineral content was also slightly (but not significantly) higher in the Mg group (Carpenter et al. 2006). In addition to the supplementation of Mg only, the mix with other minerals has also proven effective in the development and mineralization of bone in pre-adolescent girls. This is what emerges from a study conducted in Salt Lake City, Utah, in which 81 girls, with an average age of 12 years, were divided into 2 groups for 1 year: a group of 38 girls hired a chewable vitamin/mineral supplement, with a dosage of 4 cp/day, as follows: 800 mg/day of elemental Calcium (as calcium citrate and carbonate), 400 mg/day of elemental Mg (as Mg citrate and oxide) and 400 IU/day of vitamin D3, while the other group of 43 girls took a supplement (placebo) without vitamins or minerals. Girls in the supplement group showed a net gain in trabecular bone mineral density of 1.41% from baseline, while girls in the placebo group showed a net, statistically significant decline of -0.94%. Furthermore, the increase in% of the trabecular bone mineral content, after 12 months of integration, was greater in the supplemented group compared to the placebo group (5.83% versus 0.69% respectively) (Wood and Mckinnon 2001).

In conclusion, considering the studies published to date on supplementation with Mg, most have used this mineral in the form of citrate, carbonate or oxide, with a dosage varying between 250 and 1800 mg, therefore beyond UL. In all studies there was a benefit both in terms of bone mineral density and fracture risk.

## Conclusions

The recommended intake levels (RDA) of Mg were provided by the United States Food and Nutrition Board (Food and Nutrition Board 1997). These values are generally higher in men than women, and in certain situations, such as pregnancy or breastfeeding. More precisely, in the adult man the reference value varies from 400 to 430 mg/day, while in the adult woman the reference value varies from 310 to 320 mg/day. The tolerable upper intake level (UL), in relation to pharmacological contributions or supplementation, is defined for all categories except for the age groups 6–12 months and 1–3 years (for lack of experimental evidence) with a value of 250 mg/day, according to the recommendations of the EFSA document (European Food Safety Authority 2006).

From the various studies carried out since 2009 on the serum concentration of Mg and its relationship with the bone, it has been shown that lower values are related to the presence of osteoporosis, and that about 30–40% of the subjects analyzed (mainly menopausal women) have hypomagnesaemia.

Various dietetic investigations carried out have shown that many people (about 20%) constantly consume lower quantities of Mg than recommended; moreover, in this category, a lower bone mineral density and a higher fracturing risk have been found several times.

Considering the studies published to date on supplementation with Mg, most have used this mineral in the form of citrate, carbonate or oxide, with a dosage varying between 250 and 1800 mg, therefore beyond UL. In all studies there was a benefit both in terms of bone mineral density and fracture risk.

## References

[CR1] Abraham GE, Grewal H (1990). A total dietary program emphasizing magnesium instead of calcium. Effect on the mineral density of calcaneous bone in postmenopausal women on hormonal therapy. J Reprod Med.

[CR2] Abrams SA, Chen Z, Hawthorne KM (2014). Magnesium metabolism in 4-year-old to 8-year-old children. J Bone Miner Res.

[CR3] Aydin H, Deyneli O, Yavuz D (2010). Short-term oral magnesium supplementation suppresses bone turnover in postmenopausal osteoporotic women. Biol Trace Elem Res.

[CR4] Carnovale E, Marletta L (2013) Tabelle di composizione degli alimenti

[CR5] Carpenter TO, DeLucia MC, Zhang JH (2006). A randomized controlled study of effects of dietary magnesium oxide supplementation on bone mineral content in healthy girls. J Clin Endocrinol Metab.

[CR6] Castiglioni S, Cazzaniga A, Albisetti W, Maier JAM (2013). Magnesium and osteoporosis: Current state of knowledge and future research directions. Nutrients.

[CR7] Coudray C, Demigné C, Rayssiguier Y (2003). Effects of Dietary Fibers on Magnesium Absorption in Animals and Humans. J Nutr.

[CR8] Coudray C, Rambeau M, Feillet-Coudray C (2005). Dietary inulin intake and age can significantly affect intestinal absorption of calcium and magnesium in rats: a stable isotope approach. Nutr J.

[CR9] Dilworth LL, Omoruyi FO, Asemota HN (2007). In vitro availability of some essential minerals in commonly eaten processed and unprocessed Caribbean tuber crops. Biometals.

[CR10] Dimai HP, Porta S, Wirnsberger G (1998). Daily oral magnesium supplementation suppresses bone turnover in young adult males. J Clin Endocrinol Metab.

[CR11] Egger M, Dickersin K, Smith GD (2008). Problems and Limitations in Conducting Systematic Reviews. Systematic Reviews in Health Care.

[CR12] Eisinger J, Clairet D (1993). Effects of silicon, fluoride, etidronate and magnesium on bone mineral density: a retrospective study. Magnes Res.

[CR13] Elshal MF, Bernawi AE, Al-Ghamdy MA, Jalal JA (2012). The association of bone mineral density and parathyroid hormone with serum magnesium in adult patients with sickle-cell anaemia. Arch Med Sci.

[CR14] Erem S, Atfi A, Razzaque MS (2019) Anabolic effects of vitamin D and magnesium in aging bone. J Steroid Biochem Mol Biol 193 10.1016/j.jsbmb.2019.105400 31175968

[CR15] Esterle L, Sabatier J-P, Guillon-Metz F (2009). Milk, rather than other foods, is associated with vertebral bone mass and circulating IGF-1 in female adolescents. Osteoporos Int.

[CR16] European Food Safety Authority (2006) Tolerable upper intake levels for vitamins and minerals. Scientific Committee on Food; Scientific Panel on Dietetic Products, Nutrition and Allergies

[CR17] European Food Safety Authority (2009). Scientific Opinion on the substantiation of health claims related to magnesium and electrolyte balance (ID 238), energy-yielding metabolism (ID 240, 247, 248), neurotransmission and muscle contraction including heart muscle (ID 241, 242), cell division. EFSA J.

[CR18] Farrell VA, Harris M, Lohman TG (2009). Comparison between dietary assessment methods for determining associations between nutrient intakes and bone mineral density in postmenopausal women. J Am Diet Assoc.

[CR19] Food and Nutrition Board (1997) Dietary reference intakes for calcium, phosphorus, magnesium, vitamin D, and fluoride 23115811

[CR20] Gunn CA, Weber JL, Kruger MC (2014). Diet, weight, cytokines and bone health in postmenopausal women. J Nutr Health Aging.

[CR21] Hayhoe RPG, Lentjes MAH, Luben RN (2015). Dietary magnesium and potassium intakes and circulating magnesium are associated with heel bone ultrasound attenuation and osteoporotic fracture risk in the EPIC-Norfolk cohort study. Am J Clin Nutr.

[CR22] Huang J-H, Cheng F-C, Wu H-C (2015). Low magnesium exacerbates osteoporosis in chronic kidney disease patients with diabetes. Int J Endocrinol.

[CR23] Kim M-H, Yeon J-Y, Choi M-K, Bae YJ (2011). Evaluation of magnesium intake and its relation with bone quality in healthy young Korean women. Biol Trace Elem Res.

[CR24] Kunutsor SK, Whitehouse MR, Blom AW, Laukkanen JA (2017). Low serum magnesium levels are associated with increased risk of fractures: a long-term prospective cohort study. Eur J Epidemiol.

[CR25] Leclercq C, Arcella D, Piccinelli R (2009). The italian national food consumption survey INRAN-SCAI 2005–06: main results in terms of food consumption. Public Health Nutr.

[CR26] Lönnerdal B (1997). Effects of milk and milk components on calcium, magnesium, and trace element absorption during infancy. Physiol Rev.

[CR27] Mahdavi-Roshan M, Ebrahimi M, Ebrahimi A (2015) Copper, magnesium, zinc and calcium status in osteopenic and osteoporotic post-menopausal women. Clin Cases Miner Bone Metab 12:18–21. https://doi.org/10.11138/ccmbm/2015.12.1.018 10.11138/ccmbm/2015.12.1.018 PMC4469220 26136790

[CR28] Matias CN, Santos DA, Monteiro CP (2012). Magnesium intake mediates the association between bone mineral density and lean soft tissue in elite swimmers. Magnes Res.

[CR29] Mederle OA, Balas M, Ioanoviciu SD (2018). Correlations between bone turnover markers, serum magnesium and bone mass density in postmenopausal osteoporosis. Clin Interv Aging.

[CR30] Navarro-González JF, Mora-Fernández C, García-Pérez J (2009). Clinical implications of disordered magnesium homeostasis in chronic renal failure and dialysis. Semin Dial.

[CR31] Okyay E, Ertugrul C, Acar B (2013). Comparative evaluation of serum levels of main minerals and postmenopausal osteoporosis. Maturitas.

[CR32] Orchard TS, Larson JC, Alghothani N (2014). Magnesium intake, bone mineral density, and fractures: results from the Women’s Health Initiative Observational Study. Am J Clin Nutr.

[CR33] Rai SP, Sharma R (2016). Magnesium as an important marker in post-menopausal women with osteoporosis and osteopenia. Int J Pharm Sci Invent ISSN.

[CR34] Razzaque MS (2018). Magnesium: are we consuming enough?. Nutrients.

[CR35] Roth P, Werner E (1979). Intestinal absorption of magnesium in man. Int J Appl Radiat Isot.

[CR36] Seelig M (1981). Magnesium requirements in human nutrition. Magnes Bull.

[CR37] Sharma R, Sharma P, Kumar P, Gupta G (2016). Role of magnesium in post-menopausal women with osteoporosis and osteopenia. Asian J Pharm Clin Res.

[CR38] Stendig-Lindberg G, Tepper R, Leichter I (1993). Trabecular bone density in a two year controlled trial of peroral magnesium in osteoporosis. Magnes Res.

[CR39] Teo K, Chow CK, Vaz M (2009). The Prospective Urban Rural Epidemiology (PURE) study: examining the impact of societal influences on chronic noncommunicable diseases in low-, middle-, and high-income countries. Am Heart J.

[CR40] US Department of Agriculture (2009) Nutrient Database for Standard Reference, Realase 22

[CR41] US Department of Agriculture (2019) Usual Nutrient Intake from Food and Beverages, by Gender and Age, What We Eat in America, NHANES 2013–2016

[CR42] Uwitonze AM, Razzaque MS (2018). Role of magnesium in vitamin d activation and function. J Am Osteopath Assoc.

[CR43] Veronese N, Stubbs B, Solmi M (2017). Dietary magnesium intake and fracture risk: Data from a large prospective study. Br J Nutr.

[CR44] Welch AA, Skinner J, Hickson M (2017). Dietary magnesium may be protective for aging of bone and skeletal muscle in middle and younger older age men and women: cross-sectional findings from the uk biobank cohort. Nutrients.

[CR45] Wood T, Mckinnon T (2001) Calcium-Magnesium-Vitamin D Supplementation Improves Bone Mineralization in Preadolescent Girls

[CR46] Wright HH, Kruger MC, Schutte WD (2019). Magnesium intake predicts bone turnover in postmenopausal Black South African women. Nutrients.

